# Achieving Pregnancy After Early Hormone Receptor-Positive Breast Cancer: Recent Evidence and Clinical Considerations

**DOI:** 10.3390/cancers18020348

**Published:** 2026-01-22

**Authors:** Karine E. Ronan, Janice M. Walshe

**Affiliations:** 1 Princess Margaret Cancer Centre, 610 University Avenue, Toronto, ON M5G 2M9, Canada; karine.ronan@uhn.ca; 2 St. Vincent’s University Hospital, Elm Park, D04 T6F4 Dublin, Ireland

**Keywords:** breast cancer, pregnancy, endocrine therapy

## Abstract

An increasing number of young women with hormone receptor-positive (HR+) early breast cancer desire pregnancy after treatment. Prolonged adjuvant endocrine therapy, concerns regarding recurrence risk, and treatment-related fertility decline have historically complicated reproductive decision-making in this population. This narrative review synthesizes current evidence on pregnancy after early HR+ breast cancer, with particular emphasis on prospective data from the POSITIVE trial. We examine the safety of temporary endocrine therapy interruption, the impact of assisted reproductive technologies (ART) in achieving pregnancy, breastfeeding feasibility and impact, hormonal predictors of fertility, pregnancy outcomes and considerations for special populations, including BRCA mutation carriers. Retrospective studies have suggested no adverse survival impact associated with pregnancy after breast cancer. The POSITIVE trial provides prospective evidence that temporary interruption of endocrine therapy to attempt pregnancy does not increase short-term recurrence risk in selected patients. Approximately three-quarters of participants achieved pregnancy. Fertility preservation and ART were commonly used and were not associated with worse short-term oncologic outcomes. Biomarkers such as anti-Müllerian hormone offer supportive but imperfect prediction of fertility potential. Breastfeeding was feasible for many women and did not adversely affect breast cancer outcomes. Available data among BRCA mutation carriers are reassuring but largely observational. Current evidence supports the safety and feasibility of pregnancy after early HR+ breast cancer in carefully selected patients. However, longer follow-up, inclusion of higher-risk populations, and evaluation of newer therapies are needed. Individualized, multidisciplinary counselling remains central to informed decision-making.

## Simple Summary

Breast cancer is increasingly diagnosed in young women who have not yet completed their families. Historically, pregnancy after breast cancer was discouraged due to concerns about recurrence risk and the impact of the interruption of adjuvant therapy. This review summarizes the current evidence and considerations on achieving pregnancy following a diagnosis of hormone receptor-positive breast cancer, with a focus on findings from the prospective POSITIVE trial. Available data show that temporary interruption of endocrine therapy to attempt pregnancy is feasible and does not increase short-term recurrence risk in carefully selected patients. Pregnancy and live birth rates are comparable to those of the general population, and the use of fertility preservation and assisted reproductive technologies appears safe in the short term. Breastfeeding is also achievable for many women and does not appear to adversely affect outcomes. While these findings are reassuring, uncertainties remain regarding long-term safety, the impact of a break in endocrine therapy in women with higher-risk disease, BRCA mutation carriers, and new standard of care adjuvant therapies. Individualized counselling remains essential.

## 1. Introduction

Breast cancer is the most common malignancy in women of childbearing age, and its incidence in women ≤45 years of age continues to rise [1,2]. Survival outcomes among young patients have improved, with 5-year survival rates of approximately 96% for localized disease and 87% for locally advanced disease [2].

At the same time, childbearing is increasingly delayed in developed countries, with the mean age at first birth approaching 30 years and a marked rise in births among women in their 40s in the United States and Europe [3,4,5,6,7]. As a result, a growing number of women are diagnosed with breast cancer while still planning future pregnancy, with up to one-third expressing this desire at diagnosis [8].

The ability to preserve reproductive potential is often central to quality of life for many young women and may influence treatment decisions, sometimes leading patients to delay, modify, or decline recommended therapy [8,9,10,11,12]. The possibility of pregnancy can be a powerful source of hope and normalcy after treatment for many women [9]. This diversity of attitudes and perceptions underscores the complexity of fertility and childbearing decisions for young breast cancer survivors and the importance of thoughtful, individualized discussions in clinical care.

Young women often present with higher-risk disease and therefore more frequently receive (neo)adjuvant multi-agent chemotherapy and extended endocrine therapy—both of which may compromise fertility [13]. Chemotherapy can lead to ovarian failure or diminished ovarian reserve, while endocrine therapy (tamoxifen, aromatase inhibitors) and ovarian suppression (gonadotropin-releasing hormone agonists/antagonists) in hormone receptor-positive (HR+) disease further complicate conception efforts [14,15]. Historically, pregnancy was discouraged until completion of endocrine therapy due to concerns regarding recurrence, contributing to age-related fertility decline [16].

The POSITIVE trial has provided important prospective evidence supporting the short-term safety of a temporary interruption of endocrine therapy to attempt pregnancy, as well as reassuring data on pregnancy outcomes among women treated for stage I–III HR+ breast cancer [17]. Nevertheless, uncertainties remain regarding fertility potential, optimal timing, and pregnancy-related risks for some young survivors.

As increasing numbers of women consider interrupting endocrine therapy to conceive following the results of the POSITIVE trial, clear communication and individualized counselling are essential. This review synthesizes currently available evidence on pregnancy after early HR+ breast cancer, with emphasis on the recent and emerging findings from the POSITIVE trial. We explore pregnancy rates, use of assisted reproductive technology, freedom to breastfeed, biochemical predictors of fertility, and considerations around pregnancy in special populations such as carriers of germline BRCA variants, after a diagnosis of stage I–III HR+ breast cancer.

## 2. Studies Carried out in This Population

Until recently, evidence on pregnancy after breast cancer was drawn largely from retrospective studies, which consistently demonstrated no adverse effect of pregnancy on survival. Comparable outcomes were observed among women aged <40 years who conceived within 40 weeks of breast cancer diagnosis compared with those who did not become pregnant in a large Scottish cohort [18]. Similarly, a meta-analysis of 7505 young women who conceived after breast cancer treatment found no survival detriment compared with women who did not conceive [19]. Despite this evidence, small numbers of women with breast cancer conceive after treatment [20] and many women remain concerned about the safety of pregnancy after breast cancer treatment, both for themselves and their offspring [10,21].

The POSITIVE trial—a prospective, international, multicentre, single-arm study conducted by the International Breast Cancer Study Group—enrolled women aged ≤42 years with stage I–III HR+ breast cancer who had completed 18–30 months of adjuvant endocrine therapy. Participants were permitted to pause endocrine therapy for up to 2 years to pursue pregnancy, delivery, and breastfeeding, and were strongly encouraged to resume therapy afterward to complete the planned 5–10 years.

After a median follow-up of 71 months, 75.8% (n = 377) of participants achieved at least one pregnancy, and 69% had at least one live birth [22]. As expected, pregnancy declined with advancing age. In women <35 years, 85.7% conceived, compared with 76% of those aged 35–39 and 52.7% of those aged 40–42 years. The time to pregnancy increased with age also [23]. These outcomes approximate those of similarly aged women in the general population. For example, a Danish cohort reported a conception probability of 87% among women aged 30–34 and 72% among those aged 35–40 within 12 menstrual cycles [24].

Importantly, a temporary interruption of endocrine therapy did not increase short-term recurrence risk. The 5-year cumulative incidence of breast cancer events (local/regional/distant recurrence or new contralateral breast cancer) was 12.3% in POSITIVE versus 13.2% in the SOFT/TEXT control cohorts [−0.9% difference (95% CI: −4.2% to 2.6%)], and distant recurrence rates were also comparable (6.2% vs. 8.3%) [−2.1% difference (95% CI: −4.5% to 0.4%)] [22]. An 18-month landmark analysis confirmed that patients who had a pregnancy had similar breast cancer-free interval (BCFI) events compared to those who did not. As a first-of-its-kind prospective trial investigating outcomes among women who pursue pregnancy after HR+ breast cancer, this trial makes a significant contribution of new data to the field. It addresses the important question of the safety of interrupting endocrine therapy and aims to facilitate enhanced quality of life and increase pregnancy rates among young women who desire pregnancy after HR+ breast cancer. Table 1 summarizes data from large studies evaluating outcomes for women who achieve pregnancy after early breast cancer, including the POSITIVE trial.

## 3. Assisted Reproductive Technology

Between 19% and 42% of women aged ≤40 years undergo fertility preservation procedures before starting systemic treatment for breast cancer [23,25]. These practices vary, largely reflecting differences in access to reproductive services. Evidence on the safety and long-term oncologic outcomes of these interventions has been limited. Ovarian stimulation for embryo/oocyte cryopreservation at diagnosis or for IVF after cancer treatment involves the administration of gonadotropins for 10 to 14 days. This results in increased circulating estradiol levels, with concern that this may be associated with a detrimental impact on recurrence risk for women with HR+ breast cancer. In a small prospective study of women with HR+ breast cancer who underwent ovarian stimulation with gonadotropins in combination with letrozole, to minimize estradiol surge, the 5-year recurrence risk was comparable to those who did not undergo the same procedure [26].

Secondary analyses from the POSITIVE trial provide the largest prospective data on the safety of ovarian stimulation in HR+ breast cancer. Among 497 women evaluable for pregnancy outcomes, 51% pursued fertility preservation. This included 179 women (36%) who underwent ovarian stimulation for oocyte or embryo cryopreservation, 67 women (13%) who received gonadotropin-releasing hormone agonists (GnRHa) during chemotherapy, and 30 women (6%) who pursued ovarian tissue cryopreservation. One-third of those stimulated used letrozole with gonadotropins [23]. Of the 179 women who completed ovarian stimulation, 38% later transferred cryopreserved embryos [23]. At 5 years, the cumulative incidence of BCFI events was 14.0% (95% CI, 9.6–20.2%) in women who underwent ovarian stimulation and 11.5% (95% CI, 8.4–15.7%) in those who did not [22].

The use of assisted reproductive technology (ART) after study enrollment was also explored. A total of 215 patients (43%) underwent ART after enrollment, including IVF stimulation (16%), embryo transfer (14%), intrauterine insemination (7%), clomiphene (4%), embryo/egg donation (3%), and ovarian transplantation (<1%). In a 24-month landmark analysis, BCFI outcomes were similar between those who underwent ovarian stimulation for IVF and those who did not [23].

Of all of the ART methods used by patients in the POSITIVE trial, cryopreserved embryo transfer was associated with a higher chance of achieving pregnancy [23]. Over two-thirds of patients who underwent IVF after trial enrolment achieved a pregnancy [23].

These findings suggest that fertility preservation procedures and the subsequent use of ART are common among young women pursuing pregnancy after HR+ breast cancer, and although early outcomes appear reassuring, longer-term follow-up and data from larger groups of women is needed to fully assess oncologic safety.

## 4. Hormonal Factors Predictive of Fertility

Standard (neo)adjuvant chemotherapy regimens for early HR+ breast cancer often include anthracyclines, alkylating agents, and taxanes, all of which carry a risk of premature ovarian insufficiency (POI) [27]. Timed assessments of anti-Müllerian hormone (AMH), follicle-stimulating hormone (FSH), estradiol, and progesterone may provide insight into ovarian reserve and reproductive potential when interpreted alongside age and prior systemic therapy exposure [28]. As a secondary endpoint of the POSITIVE trial, hormonal analyses were used to evaluate the risk of low ovarian reserve and POI following endocrine therapy interruption. This analysis also aimed to explore associations between AMH and FSH levels and pregnancy outcomes [28].

At trial enrollment, 53% (n = 273) of participants reported amenorrhea; however 96% of the overall POSITIVE population resumed menstruation within 12 months [23]. Although prior chemotherapy was associated with delayed menstrual recovery at 6 months, no significant association was found between the time to menses resumption and chemotherapy exposure, GnRH analogue use, or age [23]. Nonetheless, menstrual regularity is a crude marker of ovarian function as many women with low ovarian reserve continue to have regular cycles [29].

AMH is a commonly used surrogate for ovarian reserve. Low levels reflect diminished reserve, and are frequently seen after breast cancer treatment [28,30]. In POSITIVE, nearly half of participants had low ovarian reserve, defined as AMH <0.5 ng/mL at month 3. This was associated with older age and prior chemotherapy, but not with the type or duration of endocrine therapy [28]. Women with low ovarian reserve had lower pregnancy rates (65% vs. 74% overall) [17].

There was no significant interaction between ART and low ovarian reserve on pregnancy success [28], although this may be biassed by the fact that patients in the study were trying to conceive in a timely manner and may have used cryopreserved material even when spontaneous conception may have been possible.

The incidence of POI (defined as FSH > 25 IU/L at month 12) among non-pregnant women was 10.6%, and all affected women had received prior chemotherapy. Elevated FSH at month 3 showed a modest association with POI development [28].

Other patient factors, such as elevated BMI, have the potential to negatively affect fertility [31]. Regional differences in BMI were observed among POSITIVE participants [32], but the effect of obesity on pregnancy outcomes was not reported.

Taken together, hormonal biomarkers offer supportive but imperfect predictive value for fertility outcomes. While serial AMH and FSH testing may assist counselling and ART referral, their role remains evolving. Approaches less dependent on ovarian reserve, including use of cryopreserved embryos or donor oocytes, may be appropriate in selected cases. Continued data collection on pregnancy following breast cancer treatment, along with the inclusion of fertility-related outcomes in clinical trial reporting, will enhance understanding and interpretation in this area.

## 5. Breastfeeding

Breastfeeding provides well-established benefits for infants and is an important consideration for mothers after breast cancer treatment. Historically, evidence on the feasibility and safety of breastfeeding in this setting has been limited, largely derived from small quantitative analyses and retrospective studies [33,34]. These studies have reported that breastfeeding is feasible for many breast cancer survivors, with no observed increased recurrence risk among the small groups of women studied. Surgery and radiotherapy for breast cancer can affect breast enlargement during pregnancy and lead to reduced milk production from the treated breast [35,36,37].

The POSITIVE trial represents the largest prospective evaluation of this issue, reporting breastfeeding outcomes as a secondary endpoint. Among the 313 participants who had at least one live birth and one intact breast, 62.6% were able to breastfeed. Notably, among women who had undergone breast-conserving surgery, 69.2% breastfed using the unaffected breast. The median duration of breastfeeding was 4.4 months [38].

At 24 months after breastfeeding initiation, BCFI events occurred in 3.6% of women who breastfed and 3.1% of those who did not, corresponding to an absolute difference of 0.5% (95% CI, −4.3% to 5.2%). Although breastfeeding decisions are influenced by multiple clinical and personal factors, these findings provide important prospective evidence to support counselling women who wish to breastfeed after breast cancer, in an area where previous data were limited [38]. Again, longer-term follow-up will be important to provide more robust safety data.

## 6. BRCA Population

Germline BRCA mutations are detected in approximately 12% of women diagnosed with breast cancer before the age of 40 years [39]. Approximately 45% of breast cancers arising in young BRCA mutation carriers are HR+ [40]. Given the distinct and often aggressive phenotype associated with BRCA-related cancers, mutation status has important implications for both treatment selection and reproductive planning [41]. Fertility considerations are particularly complex among these young women, due to a number of factors, which can include a fear of passing the pathogenic variant to offspring [42] and also the frequent recommendation to undergo risk-reducing gynecological surgery to mitigate against future malignancy [43].

Most evidence regarding the safety of pregnancy after breast cancer in BRCA mutation carriers comes from a large international retrospective cohort study of 4732 BRCA carriers diagnosed with stage I–III breast cancer at ≤40 years between January 2000 and December 2020. Among these, 659 women became pregnant after breast cancer; one-third of whom had HR+ disease. The median time from breast cancer diagnosis to conception was 4.3 years among women in the HR+ subgroup. ART was used to achieve pregnancy in 20.8% of the overall group. After a median follow-up of almost 8 years, there was no significant difference in disease-free survival observed between patients with pregnancy after breast cancer and those without (adjust HR 0.99; 95% CI 0.81–1.20) [44].

Breastfeeding safety among BRCA mutation carriers was also evaluated in this cohort. The 7-year cumulative incidence of locoregional recurrence and/or contralateral breast cancer was comparable between women who breastfed after pregnancy (n = 110) and those who did not, at 29% (95% CI, 20–40) and 36% (95% CI, 23–49), respectively. Among the women who breastfed, 36 had a prior diagnosis of hormone receptor-positive breast cancer. No statistically significant interaction was observed between breastfeeding status and hormone receptor status in analyses of locoregional recurrence and/or contralateral breast cancer incidence [34].

Fifty-four percent of the patients enrolled in the POSITIVE trial underwent BRCA mutation testing (54.1% in Europe, 76.9% in North America, 21.4% in Asia). Among those tested, 13.6% were found to carry a BRCA1/2 mutation (12.3%, 16.7%, 11.1% by region, respectively) [32]. In this small subgroup, the 3-year cumulative incidence of breast cancer events was 14.5% [17]. The POSITIVE trial did not report specific outcomes regarding the safety of ART in women with HR+ breast cancer and germline BRCA mutations [23]. The characteristics and outcomes of patients with BRCA 1/2 mutations who became pregnant after HR+ breast cancer and included in the above studies are summarized in Table 2.

The use of assisted reproductive technologies (ART) in young women harbouring pathogenic germline BRCA1/2 mutations to achieve pregnancy after breast cancer was evaluated in a retrospective cohort study including patients from Europe and Israel. Among 168 women with a BRCA mutation who conceived after breast cancer, 22 used ART. A disease-free survival (DFS) event occurred in 9.1% of women in the ART group compared with 27.4% in the non-ART group, after a median follow-up of 3.4 and 5.0 years, respectively. Notably, a higher proportion of women in the ART group had a prior diagnosis of hormone receptor-positive (HR+) breast cancer (59.1%) compared with the non-ART group (31.5%). Differences in tumour subtype distribution, together with the shorter median follow-up in the ART group, may partly explain the observed differences in DFS events; however, no concerning safety signals related to ART exposure were identified in this cohort [45].

Although these findings are reassuring and provide important information for counselling young BRCA mutation carriers, the observational nature of the data and potential residual confounding limit the extent to which definitive conclusions can be drawn.

## 7. Challenges with the Current Data

Despite the important evidence provided by the POSITIVE trial, several uncertainties remain regarding pregnancy after HR+ breast cancer. A key limitation of the trial is the under-representation of higher-risk disease. Regional lymph node involvement occurs in approximately 37–43% of cases of breast cancer diagnosed aged <45 years [2]. In the POSITIVE trial, 33.8% had lymph node-positive disease, with just 4.5% having ≥4 involved nodes. This is substantially lower than the comparative arm (the SOFT/TEXT cohorts), in which 46.6% were lymph node-positive and 11.7% had ≥4 positive nodes [17]. As a result, the safety of interrupting endocrine therapy for women with higher-risk disease, particularly those with ≥4 involved nodes, cannot be inferred from the POSITIVE trial. Furthermore, young women are disproportionately diagnosed with triple-negative or HER2-positive breast cancer. The POSITIVE trial exclusively evaluated HR+ disease and does not inform pregnancy safety or timing for these breast cancer subtypes.

Adjuvant chemotherapy has a proven benefit for premenopausal women with intermediate genomic risk or intermediate-to-high clinical risk breast cancer [46]. In the POSITIVE trial, 62% of participants received (neo)adjuvant chemotherapy, compared with 76.1% in the SOFT/TEXT control group. This lower rate of chemotherapy reflects the more favourable risk profile of the POSITIVE population and limits the generalizability of its findings—particularly regarding gonadotoxic drug exposure and long-term oncologic outcomes in higher-risk young women.

Notably, 25.9% of POSITIVE participants had HER2-positive disease [32] (compared with 22.9% and 22.2% in SOFT and TEXT, respectively [47]), a large proportion of whom would be recommended chemotherapy on this basis. This indicates that a meaningful proportion of the chemotherapy-treated cohort had HER2-positive, rather than higher-risk HR+ disease, further underscoring the lower-risk HR+ profile of the POSITIVE population.

Although the available short-term safety data on endocrine therapy interruption are reassuring [22], longer follow-up is essential, particularly for HR+ disease, which carries a well-characterized risk of late recurrence. Emerging data from POSITIVE also highlight that fertility preservation procedures and ART appear feasible and increasingly utilized, although their long-term oncologic safety remains incompletely defined, particularly among higher-risk subgroups and BRCA mutation carriers [23]. Hormonal biomarkers such as AMH and FSH offer some insight into ovarian reserve and premature ovarian insufficiency after breast cancer treatment but remain imperfect predictors of successful conception [28]. Their role is best considered as an adjunct to reproductive counselling which should also consider other factors that determine likelihood of pregnancy including patient age and prior chemotherapy treatment. The POSITIVE trial has provided reassurance that neither the type nor duration of endocrine therapy was associated with impaired fertility [28]. Evidence regarding breastfeeding after endocrine therapy interruption is also reassuring, with longer follow-up required for all exploratory endpoints to confirm long-term safety [38].

Finally, while POSITIVE enrolled participants from multiple regions, representation across racial and ethnic groups was uneven. Women of Asian origin (Japan and South Korea) comprised 14.1% of the cohort, whereas African American (1.4%) and Middle Eastern (0.6%) women were markedly underrepresented [32]. This limits the ability to assess outcomes across diverse populations.

## 8. Novel Systemic Therapies

Since the POSITIVE trial completed enrolment, treatment standards for HR+ breast cancer have evolved. Adjuvant CDK4/6 inhibitors have become standard for many patients with higher-risk HR+ disease, demonstrating improvements in disease-free survival and signals toward overall survival benefit [48,49]. Since adjuvant abemaciclib was first approved by the F.D.A in late 2021, followed by the approval of adjuvant ribociclib in 2024, no participants in the POSITIVE trial received adjuvant CDK4/6 inhibition. The effects of these agents on fertility remain unknown, and it is unclear whether temporary interruption of therapy is compatible with their intended use, particularly as adjuvant ribociclib is recommended for 3 years [49]. Given that all node-positive patients—and node-negative patients with high-risk features—may now be considered eligible for CDK4/6 inhibitors (in jurisdictions where they are available), these uncertainties apply to a substantial proportion of women similar to those enrolled in the POSITIVE trial.

Data regarding the potential impact of CDK4/6 inhibitors on fertility is very limited. By inhibiting key regulators of cell cycle progression, CDK inhibitors may impact ovarian tissue and potentially lead to gonadotoxicity [50].

An exploratory analysis of the PENELOPE-B trial measured hormonal factors (estradiol, FSH, and AMH) in premenopausal women at baseline, cycle 7 of treatment, and at 30 days post-completion of treatment (EOT) in an attempt to measure ovarian function and fertility [51]. While no difference in hormonal factors was observed between the palbociclib and placebo groups in the overall population, among patients aged under 40 years, 28.1% in the palbociclib arm and 24.7% in the placebo arm had postmenopausal hormone levels (FSH > 12.4 IU/L and estradiol < 52.2 ng/L) at baseline, versus 27.4% and versus 14.5% by the end of treatment. Rates of non-fertile AMH were high at baseline in the overall group (93.6% for palbociclib and 91.7% for placebo) and did not change significantly across the three timepoints. However among women aged <40 years, non-fertile AMH levels increased from 80.9% at baseline to 86.9% at EOT, compared with 77.8% at baseline and 78.9% at EOT with placebo [51]. While neither of these differences were statistically significant, the sample size was low. These quantitative changes, however, particularly an absolute increase of 13% in postmenopausal hormone levels between the palbociclib and placebo groups at EOT, may be meaningful [52].

While low AMH in the POSITIVE trial was associated with lower pregnancy rates compared to the overall trial population, 65% of patients with low AMH levels achieved pregnancy, but a higher threshold was used to define low AMH in the POSITIVE trial compared with the PENELOPE-B analysis (<0.5 ng/mL versus <0.22 ng/mL) [28,51].

While this data is thought-provoking, whether it translates to a meaningful impact on fertility after CDK4/6 inhibitor treatment is uncertain. Dedicated research on the gonatoxic effects of CDK4/6 inhibitors is required to adequately counsel women on the potential effects of these agents, which are not well-known.

## 9. Conclusions

Despite a growing population of women previously treated for breast cancer who have not started or completed their family, pregnancy rates after breast cancer are less than half of that in age-matched women in the general population [53,54]. The POSITIVE trial represents a landmark prospective study addressing the safety and feasibility of the temporary interruption of adjuvant endocrine therapy to pursue pregnancy in young women with HR+ early breast cancer. Its findings provide important reassurance that, in carefully selected patients, treatment interruption is not associated with an increased risk of recurrence in the short term [17,22]. The longer-term risks of this approach will emerge as follow-ups continue for 10 years.

Recent evidence on ART, hormonal biomarkers, and breastfeeding from this pivotal trial represents a significant advance in counselling women about postpartum decisions after HR+ breast cancer. The psycho-oncological companion study to the POSITIVE trial is yet to be reported. This aims to evaluate psychological distress, fertility concerns, and decisional conflicts among women enrolled in POSITIVE. Its findings are expected to identify gaps in supportive care and guide the development of targeted interventions to better address the emotional and practical needs of young women pursuing pregnancy after breast cancer.

Despite the important trial output, knowledge gaps remain. Key unanswered questions include longer-term oncologic safety of endocrine therapy interruption, the integration of newer treatments into fertility considerations, and the optimal management of women with higher-risk disease or diverse ethnic and socioeconomic backgrounds who remain underrepresented in clinical trials. However, as we stand, we are richer in the knowledge that pregnancy after HR+ breast cancer is safe and possible, but decision-making remains individualized.

## Figures and Tables

**Table 1:** Studies evaluating pregnancy after HR+ early breast cancer.

Study	POSITIVE Trial [17]	*Lambertini* et al. [19]	*Anderson* et al. [18]
Study design	Prospective, multicentre, single arm	Systematic review and meta-analysis	Retrospective cohort study
Duration of follow-up	71 months [22]		
Inclusion criteria	Women aged ≤42 years; stage I–III HR+ breast cancer; had received adjuvant endocrine therapy for 18–30 months	Studies reporting on pregnancy after BC; likelihood on pregnancy after BC; reproductive outcomes; and/or maternal safety; possibility to estimate data on relative risk (RR), odds ratio (OR), and hazard ratio (HR), according to the analyzed outcome, with their 95% CIs	Women with breast cancer diagnosed at age 20–39 years between 1981 and 2017 and had a subsequent LB
HR+ only (yes/no)	Yes	No	No
Total number of pts	516	7505	290
Total number of HR+ pts	516	Data not available	102
Endocrine therapy interruption (yes/no)	Yes	Not reported	Not reported
Pregnancy outcomes: - Pregnancy rate - Live birth rate	75.8% [22] 69% [22]	60% reduced likelihood of subsequent pregnancy after BC compared with the general population (HR 0.40; 95% CI, 0.32 to 0.49)	5.7% of total study population (n = 5181)
Oncologic outcomes: - DFS - 5 yr cumulative incidence of BCFI events - OS	12.3% (vs. 13.2% in SOFT/TEXT control cohort) [22]	HR 1.10; 95% CI, 0.73 to 1.66 for pts with pregnancy after HR+ BC vs. those without	OS increased compared to those who did not have an LB, HR 0.65 (95%CI 0.50–0.85)

HR+, hormone receptor-positive; BC, breast cancer; DFS, disease-free survival; BCFI, breast cancer-free interval; OS, overall survival; LB, livebirth.

**Table 2:** Characteristics and outcomes among patients with BRCA 1/2 mutations who became pregnant after HR+ breast cancer in recent studies.

	*Lambertini* et al. [44]	POSITIVE Trial
Total no. of pts with BRCA1/2 mutation in the study	4732	38 [32]
No. of pts with BRCA1/2 mutation who underwent mastectomy	2794	30 [32]
No. of pts with BRCA1/2 mutation who became pregnant after breast cancer	659	NR
No. of pts with BRCA1/2 mutation who used ART	121	NR
No. of pts with BRCA1/2 mutation who became pregnant after **HR+** breast cancer	216	NR
No. of pts with BRCA1/2 mutation who breastfed after HR+ breast cancer	36 [34]	NR
3-year cumulative incidence of breast cancer events among women with BRCA 1/2 mutation	NR	14.5% [17]
DFS in pts with BRCA 1/2 mutation who had a pregnancy after HR+ breast cancer (vs. pts with no pregnancy)	HR 1.29 (0.98–1.70), *p* = 0.04	NR

Pts, patients; NR, not reported; ART, assisted reproductive technology; HR+, hormone receptor-positive; DFS, disease-free survival.

## Data Availability

No new data were created or analyzed in this study. Data sharing is not applicable to this article.
